# The Pan-Cancer Crosstalk Between the *EFNA* Family and Tumor Microenvironment for Prognosis and Immunotherapy of Gastric Cancer

**DOI:** 10.3389/fcell.2022.790947

**Published:** 2022-03-02

**Authors:** Rongrong Xie, Mengping Yuan, Yiyan Jiang

**Affiliations:** ^1^ Department of Radiotherapy, The First Affiliated Hospital of Wenzhou Medical University, Wenzhou, China; ^2^ Department of Gastroenterology, The Second Affiliated Hospital of Wenzhou Medical University, Wenzhou, China; ^3^ Department of Medical Oncology, The First Affiliated Hospital of Wenzhou Medical University, Wenzhou, China

**Keywords:** EFNA, gastric cancer, tumor microenvironment, immune cell infiltration, drug sensitivity, microsatellite instability, tumor mutational burden, immune checkpoint

## Abstract

**Background:** *EFNA1–5* have important physiological functions in regulating tumorigenesis and metastasis. However, correlating *EFNA* genes in the tumor immune microenvironment (TIME), and the prognosis of patients with gastric cancer remains to be determined.

**Methods:** Using public databases, the expression of *EFNA1-5* in pan-cancer and gastric cancer was comprehensively analyzed using UCSC Xena, the Oncomine dataset and UALCAN. We further completed survival analysis by Kaplan-Meier plotter to evaluate the prognosis of the high and low expression groups of the *EFNAs* gene in patients with gastric cancer. The TIMER tool was used to reveal the correlation between immune cell infiltration and genes of interest. Spearman correlation was used to find an association between the *EFNA* genes and tumor stem cells, TIME, microsatellite instability (MSI) or tumor mutational burden (TMB). We also used cBioportal, GeneMANIA and STRINGS to explore the types of changes in these genes and the protein interactions. Finally, we described the TIME based on QUANTISEQ algorithm, predicted the relationship between the *EFNA* genes and half-maximal inhibitory concentration (IC_50_), and analyzed the relationship between the *EFNA* family genes and immune checkpoints.

**Results:** The expression of *EFNA1*, *EFNA3*, *EFNA4*, and *EFNA5* was elevated in pan-cancer. Compared with normal adjacent tissues, *EFNA1*, *EFNA3*, and *EFNA4* were up-regulated in gastric cancer. In terms of the influence on the survival of patients, the expression of *EFNA3* and *EFNA4* were related to overall survival (OS) and disease-free survival (DFS) for patients with gastric cancer. High expression of *EFNA5* often predicted poor OS and DFS. In gastric cancer, the expression of *EFNA3* and *EFNA4* showed a significant negative correlation with B cells. The higher the expression of *EFNA5*, the higher the abundance of B cells, CD4+T cells and macrophages. CD8+T cells, dendritic cells infiltration and *EFNA1-4* expression were negatively correlated. The infiltration of CD4+T cells, macrophages and neutrophils was negatively correlated with the expression of *EFNA1*, *EFNA3*, and *EFNA4*. TMB and MSI were positively correlated with *EFNA3*/*EFNA4* expression. In the tumor microenvironment and drug sensitivity, *EFNA3/4/5* also showed a significant correlation. In addition, we explored the relationship between the *EFNA* family genes and the immune microenvironment (B cells, M2 macrophages, monocytes, CD8^+^ T cells, regulatory T cells, myeloid dendritic cells, natural killer cells, non-regulatory CD4^+^ T cells), immune checkpoint (*PDCD1*, *PDCD1LG2*, *CD274*, *CTLA4*), and IC_50_ of common chemotherapeutic drugs for gastric cancer (5-fluorouracil, cisplatin, docetaxel and gemcitabine).

**Conclusions:** Our study provides new ideas for tumor treatment and prognosis from the perspective of TIME, and nominates *EFNA1*–*5* to become potential therapeutic targets for gastric cancer.

## Introduction

Gastric cancer is the fifth most common malignancy and fourth in incidence worldwide (Bray et al., 2018). The benefit of chemotherapy and targeted therapy for patients with gastric cancer is still lower than that of most other cancers, with treatment failure mostly due to local recurrence, distant metastasis and drug resistance (Song et al., 2017; Biagioni et al., 2019; Kim et al., 2021). Nowadays, anti-cancer immunotherapies are emerging, including immune checkpoint inhibitors, cancer vaccines, adoptive cell transfer, cytokines, and adjuvants (da Silva et al., 2019; Fu et al., 2021). In patients with cancer, tumors often control immune checkpoints (such as CTLA-4 or PD-1/PD-L1) to cause T cell dysfunction or inhibition which blocks the host anti-tumor immune response to protect tumor tissue (Binnewies et al., 2018; Taube et al., 2018; Jia et al., 2020). The tumor microenvironment (TME), which includes immune cells, stromal cells and cancer cells, is dynamic and constantly evolving to promote tumor cell growth, metastasis and immune escape (Anderson and Simon, 2020; Bader et al., 2020; Jia et al., 2020; Lee et al., 2021). Increasing evidence reveals the important role of the TME in the biological behavior, occurrence and progression mechanism of breast cancer, gastric cancer and other tumors (Goff et al., 2021; Lee et al., 2021; Pei et al., 2021).

Erythropoietin producing hepatocyte (Eph) receptors, a large family of receptor tyrosine kinases, are expressed in most tissues during embryogenesis (Nakamura et al., 2005; Strozen et al., 2021). The Eph/Ephrin (EFN) signaling axis is a key signaling pathway in many developmental processes and an important mediator of neurogenesis, capillary budding, cell proliferation, differentiation, morphogenesis, adhesion, migration and death (Hong et al., 2018; Yin et al., 2020; Strozen et al., 2021). Eph receptors are defined as two subfamilies based on their affinity for ligands and sequence homology of extracellular domains, namely 9 Class A receptor members EphA (Epha1-8 and 10) and 5 Class B receptor members EphB (EphB1-4 and 6), for a total of 14 members in mammals (Uchiyama et al., 2015; Koh et al., 2020). These receptors bind to glycosylphosphatidylinositol-anchored ligands Ephrin-A (A1-A5) and transmembrane Ephrin-B (B1-B3) with short cytoplasmic regions containing PDZ binding motifs (Uchiyama et al., 2015). In recent years, members of this family have been investigated for their role in regulating tumorigenesis, aggressiveness, tumor-related angiogenesis, metastasis, and prognosis (Leite et al., 2020; Ieguchi and Maru, 2021). Furthermore, EFNA2 has been found to play an important role in angiogenesis and promoting epithelial-mesenchymal transformation in prostate cancer through *in vitro* and *in vivo* migration—and therefore a potential therapeutic target for prostate cancer (Zhao et al., 2021). EFNA4 is up-regulated in hepatocellular carcinoma correlating to a poor prognosis. Its overexpression mainly affects the PIK3R2/GSK3β/β -catenin pathway which significantly promotes the progression (proliferation and migration) of hepatocellular carcinoma (Lin et al., 2021). In recent years, there has also been reports on EphA1 and EphA2 in the field of gastric cancer (Rudno-Rudzińska et al., 2017; Peng C et al., 2018). Previous studies have provided new insights into anti-cancer therapies which prompted us to explore the mechanistic role of EFNA in the TME and its prognostic role in cancer.

In this study, the *EFNA* genes were analyzed and explored by bioinformatics, and the differences in transcriptional level of each *EFNA* gene in gastric cancer tissues and normal tissues were compared to evaluate its prognostic value in gastric cancer. The relationship between *EFNA* expression and immune cell infiltrates, TME, immune checkpoints, IC_50_ of common chemotherapeutic drugs, tumor mutational burden (TMB) and microsatellite instability (MSI) was also investigated.

## Materials and Methods

### Transcription Analysis With Oncomine

We used the UCSC Xena (https://xenabrowser.net/datapages/) search tool to obtain gene expression data for various primary cancers, including survival information, as well as data for RNA-sequencing (RNA-seq), immune subtypes, DNA stemness score (DNA-ss), and RNA stemness score (RNA-ss) (Goldman et al., 2020). We also used the Oncomine database, a cancer microarray website (www.oncomine.org) to query, extract tumor genes, and visualize data (Rhodes et al., 2004). The *EFNA* expression was explored in different cancers, comparing transcriptional differences of *EFNA1-5* between cancer samples and normal controls using Student t test. The significance threshold of P value was defined as 0.05.

### Identification of Differential Gene Expression With UALCAN

The UALCAN database (http://UALCAN.path.uab.edu/), a comprehensive, online, publicly accessible resource, was used to obtain RNA sequence transcriptome data from The Cancer Genome Atlas (TCGA) database (Chandrashekar et al., 2017). We used UALCAN to search for differential gene expression of *EFNA1-5* between gastric cancer tissue and normal tissue samples.

### Prognostic Analysis With Kaplan-Meier Plotter

We used Kaplan-Meier plotter, an open database (www.kmplot.com), which contains clinical information such as mRNA levels of tumor genes, prognosis, survival time and survival status of patients (Guo and He, 2020). In this study, median *EFNA* gene expression data of patients with gastric cancer were used classify them into high or low expression groups. The Kaplan-Meier survival curve was used to focus on *EFNA* expression, overall survival (OS) and disease-free survival (DFS) of patients with gastric cancer. The hazard ratio was given with a 95% confidence interval (CI), and *p* < 0.05 was considered statistically significant.

### Prediction of Chemosensitivity

From the TCGA database, tumor RNA-seq data from the Genomic Data Commons (GDC) portal was downloaded. We predicted individual chemotherapy responses based on the Genomics of Drug Sensitivity in Cancer (GDSC) (https://www.cancerrxgene.org/). The half-maximal inhibitory concentration (IC_50_) of drugs was predicted by the pRRophetic algorithm. The ridge regression model of the IC_50_ of the sample was constructed with the ‘pRRophetic’ R package. A box diagram was drawn of the difference in IC_50_ between high and low *EFNA* expression groups as determined using the Wilcoxon signed-rank test of the R v4.1.2 software.

### Changes in Patterns and Protein Interaction Analysis Using cBioPortal, GeneMANIA, and STRINGS

cBioportal (https://www.cbioportal.org/) was used for cancer genome information network platform analysis (Cerami et al., 2012). The change patterns (amplification, mutation, deletion, etc.) and proportion of *EFNA* genes were evaluated based on the TCGA database. The *EFNA* genes were submitted in GeneMANIA (http://www.genemania.org), an online research tool (Warde-Farley et al., 2010), whereby the site analyzed and displayed genes that performed similar functions—presenting an interaction between protein expression and heredity in a network. Furthermore, STRINGS (https://string-db.org/), contains vast amounts of protein-protein interaction (PPI) data (Szklarczyk et al., 2019) used to elucidate the PPI network of *EFNA1-5*.

### Correlation Between Gene Expression and Immune Cell Abundance

The TIMER resource (http://timer.cistrome.org/), an intuitive, user-friendly tool, was used to visualize immune cell abundance with various factors such as gene expression, somatic cells and the function of the relationship between clinical features (Li T. et al., 2020). We used TIMER to evaluate the relationship between *EFNA1*–*5* expression and infiltration of immune cells in gastric cancer. Besides, we also used the QUANTISEQ algorithm for depicting the tumor immune microenvironment (TIME). The immune score was evaluated by the ‘ggplot2’ and ‘pheatmap’ R packages. Lastly, we used the ‘immunedeconv’ R package which integrated six of the latest algorithms: TIMER, xCell, MCP-counter, CIBERSORT, EPIC, and quanTiseq.

### Association of Genes Expression With TIME and Stem Cell Index

The ‘ESTIMATE’ and ‘Limma’ R Packages were used to obtain the level of stromal and immune cell infiltration in various types of cancer. The Spearman method was used to explore the correlation between *EFNA* genes expression, tumor stem cells, and TIME in pan- and gastric cancer.

### Correlation Analysis Between *EFNA* Family Genes and Immune Checkpoints

To correlate the *EFNA* family genes with the immune checkpoints, we used the mRNA-seq data from the TCGA tumors (https://tcga-data.nci.nih.gov/tcga/). The two-gene correlation was analyzed with the ‘ggstatsplot’ R package, and the multi-gene correlation was analyzed using the ‘pheatmap’ R package. Spearman’s correlation analysis was used to show the correlation between quantitative variables with non-normal distribution.

### Statistical Analysis

All statistical analyses were performed using R v4.1.2 and SPSS v26.0. We used R ‘ggplot2’, ‘pheatmap’, ‘ggpubr’, ‘corrplot’ or ‘survminer’, ‘limma’, and other software packages to map and visualize data. The student’s *t*-test was used to compare the differential expression of *EFNA1-5* genes between gastric cancer and normal specimens. The log-rank test was used to compare the survival time of patients between high and low gene expression groups. The Spearman method was used to analyze the correlation between *EFNA1-5* genes and MSI/TMB. *p* < 0.05 was defined as statistically significant.

## Results

### Heterosexual Expression of *EFNA1-5* in Pan-Cancer

The results showed that *EFNA1* and *EFNA4* had the highest expression in pan-cancer, followed by *EFNA3* and *EFNA5* with high expression, and *EFNA2* with low expression (Supplementary Figure S1A). *EFNA4* had the strongest positive correlation with *EFNA3* (Cor = 0.55, Supplementary Figure S1B). On the contrary, *EFNA5* and *EFNA2* were negatively correlated with each other (Cor = −0.21, Supplementary Figure S1B). The heat map of Supplementary Figure S1C further shows that the expression of each gene in the *EFNA* is highly heterogeneous in different cancer species. The expression of *EFNA1* was high in bladder urothelial carcinoma (BLCA), *EFNA2* was highest in stomach adenocarcinoma (STAD), and *EFNA3* was highest in lung squamous cell carcinoma (LUSC). *EFNA4* was highly expressed in cholangiocarcinoma (CHOL). *EFNA5* was also highest in CHOL, but low in most other cancers.

### Transcriptional Levels of *EFNA1-5* in Gastric Cancer and Versus Healthy Tissues for Diagnosis of Gastric Cancer

**FIGURE 1:**
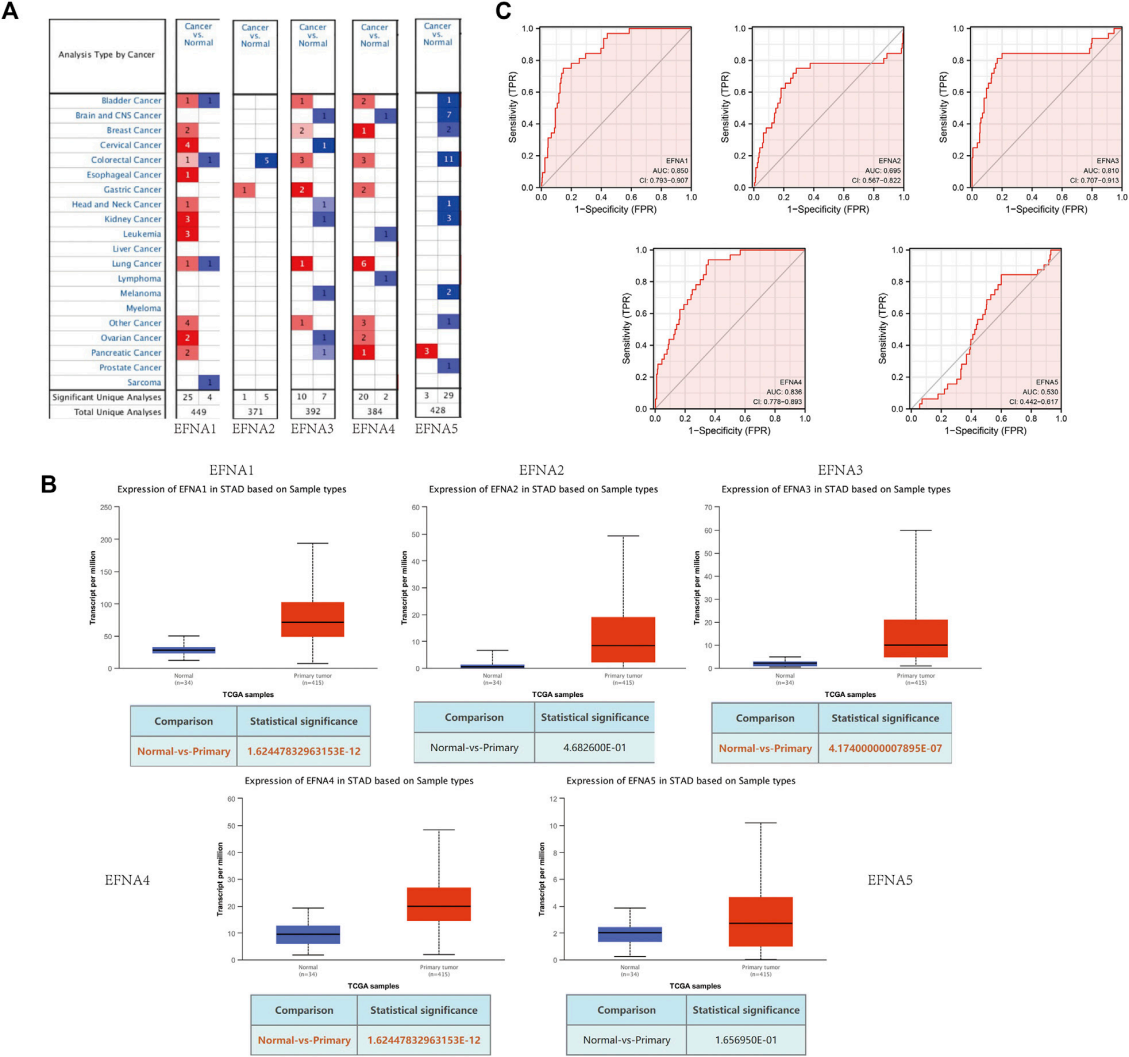
Expression of *EFNA1-5* in gastric cancer and normal tissues. **(A)** mRNA levels of *EFNA* in various cancers. Red represents up-regulated mRNA expression and blue represents down-regulated mRNA expression. **(B)** Transcription of *EFNA1-5* in gastric cancer and normal tissues from UALCAN data. **(C)** ROC curves of the *EFNA* genes.

In this study, transcription levels of *EFNA* genes in cancer and normal tissues were retrieved using the Oncomine database. From the results shown in Figure 1A, compared with normal tissues, there was an increase in transcription levels of *EFNA2*, *EFNA3*, and *EFNA4* in gastric cancer tissues.

UALCAN was used to analyze the expression pattern of *EFNA1-5* in gastric cancer and normal tissues. As shown in Figure 1B, the expression of *EFNA1* (*p* = 1.62E-12), *EFNA3* (*p* = 4.17E-07), and *EFNA4* (*p* = 1.62E-12) were significantly increased in gastric cancer tissues. However, there was no significant difference between *EFNA2* (*p* = 4.68E-01) and *EFNA5* (*p* = 1.66E-01) expression.

We evaluated the sensitivity and specificity of *EFNA* genes to distinguish between people with gastric cancer and healthy people by using a receiver operating characteristic (ROC) curve. As shown in Figure 1C, *EFNA1* (area under curve [AUC] = 0.850, CI: 0.793–0.907), *EFNA3* (AUC = 0.810, CI: 0.707–0.913), and *EFNA4* (AUC = 0.836, CI: 0.778–0.893) have high diagnostic value. *EFNA2* (AUC = 0.695, CI: 0.567–0.822) also showed a high but lower diagnostic value. In contrast, *EFNA5* (AUC = 0.530, CI: 0.442–0.617) was of moderate discriminative diagnostic value.

### Prognostic Potential of *EFNA* Genes on Survival in Gastric Cancer

**FIGURE 2:**
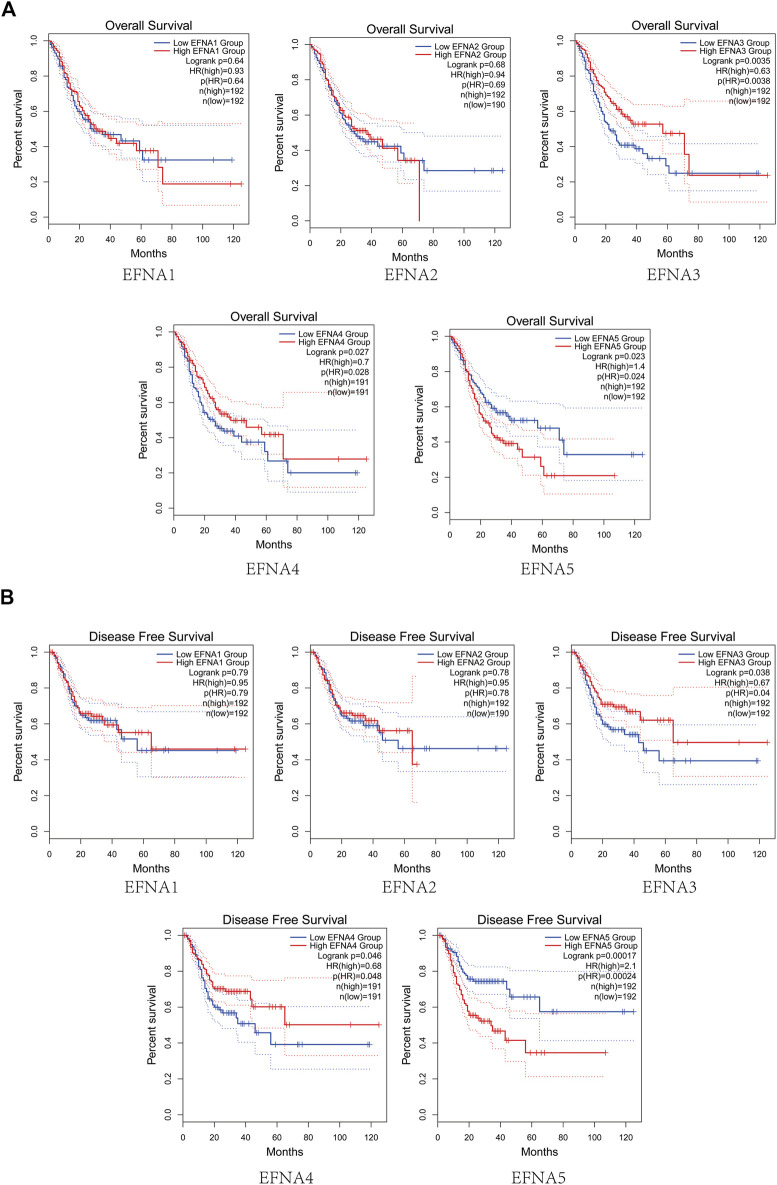
Survival analysis of gastric cancer. **(A)** Analysis curve of *EFNA* expression and overall survival rate in gastric cancer (Kaplan-Meier plotter). **(B)** Analysis curve of *EFNA* expression and disease-free survival rate in gastric cancer (Kaplan-Meier plotter).

The prognostic value of *EFNA1-5* in patients with gastric cancer for OS was evaluated. As shown in Figure 2A, the OS in the high expression group of *EFNA3* and *EFNA4* was significantly higher than that in the low expression group (*p* = 0.0035 and *p* = 0.027, respectively). On the contrary, the OS in the high expression group of *EFNA5* was significantly lower than that in the low expression group (*p* = 0.023). For *EFNA1* and *EFNA2* expression, there was no significant difference in OS between the high expression and the low expression groups. We next explored the effect of *EFNA* genes expression on DFS. As shown in Figure 2B, high expression of *EFNA3* (*p* = 0.038) and *EFNA4* (*p* = 0.046) showed longer DFS. However, high expression of *EFNA5* suggested poor DFS (*p* = 0.00017). Similarly, there was no statistical difference in DFS between the *EFNA1* and *EFNA2* expression groups.

### Relationship Between the Expression of EFNA Family Genes and the IC_50_ of Common Chemotherapeutic Drugs for Gastric Cancer

**FIGURE 3:**
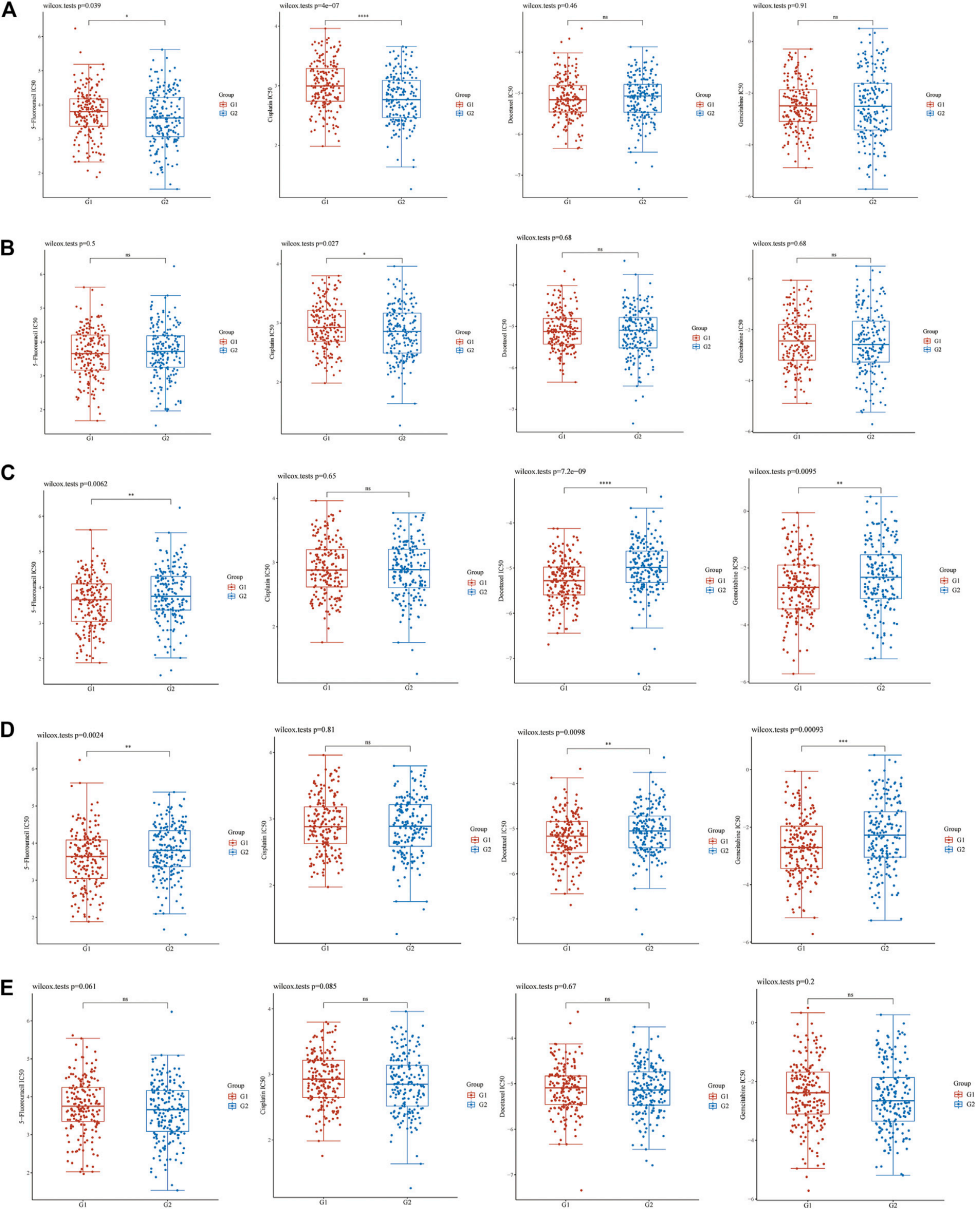
IC_50_ difference between high or low *EFNA* family genes expression of four chemotherapeutic drugs (5-fluorouracil, cisplatin, docetaxel, and gemcitabine). **(A)** *EFNA1*, **(B)** *EFNA2*, **(C)** *EFNA3*, **(D)** *EFNA4*, and **(E)** *EFNA5*. The horizontal axis represents samples of different groups, the vertical axis represents the distribution of the IC_50_ scores, the different colors represent different groups, and the upper left corner represents the significance of the *P*-value test method.

The box diagram for the differences in IC_50_ of chemotherapeutic drugs between high and low gene expression groups showed that the expression of *EFNA1* was related to the IC_50_ of 5-fluorouracil (*p* = 0.039) and cisplatin (*P* = 4E-07) (Figure 3A). The expression of *EFNA2* was also associated with IC_50_ of cisplatin (*p* = 0.027) (Figure 3B). The expressions of *EFNA3* and *EFNA4* were related to the IC_50_ of 5-fluorouracil (*p* = 0.0062 and *p* = 0.0024, respectively), docetaxel (*p* = 7.2E-09 and *p* = 0.0098, respectively), and gemcitabine (*p* = 0.0095 and *p* = 0.00093, respectively) (Figures 3C,D). However, no correlation was found between *EFNA5* expression and the IC_50_ of common gastric cancer chemotherapeutic drugs (Figure 3E).

### Drug Sensitivity Analysis of *EFNA* Genes

We used Pearson correlation analysis to study the relationship between *EFNA1-5* expression and drug sensitivity. The scatter plot showed that *EFNA3* expression was positively correlated with drug sensitivity of SR16157 (Supplementary Figure S4A, Cor = 0.488, *p* < 0.001) and fulvestrant (Supplementary Figure S4G, Cor = 0.421, *p* < 0.001). *EFNA4* expression was negatively correlated with drug sensitivity of selumetinib (Supplementary Figure S4D, Cor = –0.456, *p* < 0.001), cobimetinib (isomer 1) (Supplementary Figure S4E, Cor = −0.445, *p* < 0.001) and trametinib (Supplementary Figure S4M, Cor = −0.398, *p* = 0.002). EFNA5 expression was negatively correlated with drug sensitivity of XK-469 (Supplementary Figure S4B, Cor = −0.467, *p* < 0.001), dimethylaminoparthenolid (Supplementary Figure S4C, Cor = −0.466, *p* < 0.001), BN-2629 (Supplementary Figure S4F, Cor = −0.429, *p* < 0.001), lomustine (Supplementary Figure S4H, Cor = −0.414, *p* = 0.001), arsenic trioxide (Supplementary Figure S4I, Cor = −0.414, *p* = 0.001), homoharringtonine (Supplementary Figure S4J, Cor = −0.406, *p* = 0.001), vincristine (Supplementary Figure S4K, Cor = −0.405, *p* = 0.001), epirubicin (Supplementary Figure S4L, Cor = −0.403, *p* = 0.001), carmustine (Supplementary Figure S4N, Cor = −0.397, *p* = 0.002), and daunorubicin (Supplementary Figure S4O, Cor = −0.396, *p* = 0.002), while positively correlated with irofulven (Supplementary Figure S4P, Cor = 0.381, *p* = 0.003).

### Correlation Between of *EFNA* Genes, Gene Changes, and Protein Interactions in Gastric Cancer

**FIGURE 4:**
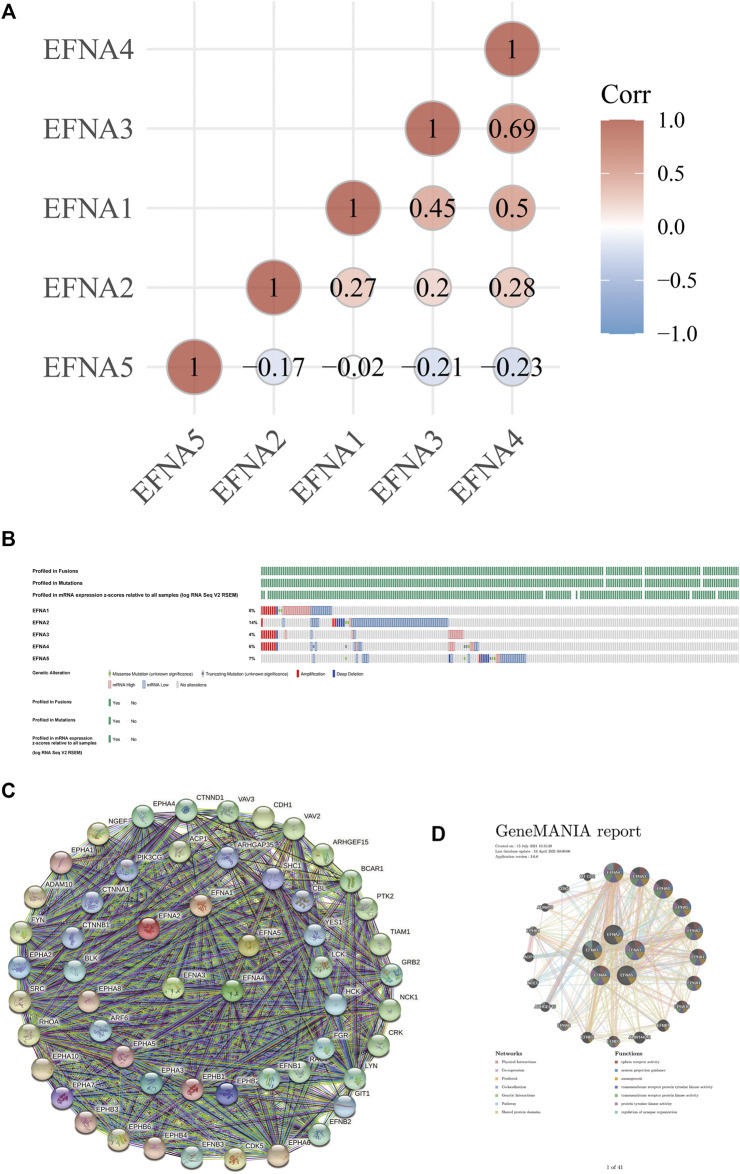
Correlation analysis of EFNA genes in gastric cancer, gene changes and protein interactions. **(A)** Correlation of different genes in between different EFNA genes in gastric cancer. **(B)** Types and proportions of EFNA gene changes in gastric cancer samples. **(C,D)** Protein interaction network of different genes in EFNA involved with different EFNA genes.

Figure 4A shows the degree of association between *EFNA* genes. Among them, the correlation between *EFNA3* and *EFNA4* was the strongest with a positive correlation. *EFNA1* also had moderate positive correlation with *EFNA3* and *EFNA4*. *EFNA2* was positively correlated with *EFNA1*, *EFNA3*, and *EFNA4*. *EFNA5* showed mild to moderate negative correlation with the other four genes.

In terms of genetic changes, we explored the regulatory effect of genetic changes on *EFNA* transcription level using data from the TCGA database. Figure 4B shows the proportion of *EFNA* genes altered in samples and the type of genes altered, which was analyzed and visualized using cBioPortal. Among the gastric cancer samples queried, the samples with changes in *EFNA1*, *EFNA2*, *EFNA3*, *EFNA4*, and *EFNA5* accounted for 8, 14, 4, 6, and 7% of the total population, respectively. Gene changes affect the expression of cancer-related genes and thus affect the occurrence and development of tumors. Genetic alterations include missense mutations, truncation mutations, deep deletions, and increased/decreased mRNA expression. The main changes related to the *EFNA1* gene were the enhancement of mRNA expression, followed by the decrease and amplification of mRNA expression. The majority of *EFNA2* gene changes were in the form of reduced mRNA expression. The gene changes of *EFNA3* were mainly concerning mRNA expression enhancement and amplification. The gene changes of *EFNA4* were associated with decreased and amplified mRNA expression, followed by enhanced mRNA expression. The *EFNA5* gene was most attenuated in mRNA expression. Overall, low mRNA expression was the most common genetic change associated with *EFNA* genes in our gastric cancer samples.

To explore the potential relationship of *EFNA* genes, GeneMANIA was used in this study to analyze the PPI network. The network diagram in Figure 4C shows 5 *EFNA* proteins and 50 proteins associated with them. We also explored the co-expression of the *EFNA* genes. Thus, the gene-gene network was constructed based on the five *EFNA* genes. GeneMANIA is available to explore gene interactions, and we used it to predict the genes that interact with gastric cancer and to build our representative interaction network. Figure 4D shows 20 nodes surrounding the central nodes of the five *EFNA* genes, which are genes associated with *EFNA* in physical interaction, co-expression, prediction, co-location, genetic interaction, pathways and shared protein domain. Among them, *EPHA4*, *EPHA3*, *EPHA8*, *EPHA5*, and *EPHA2* ranked high in correlation.

### Correlation Between *EFNA1-5* and Immune Cell Abundance in Patients With Gastric Cancer

**FIGURE 5:**
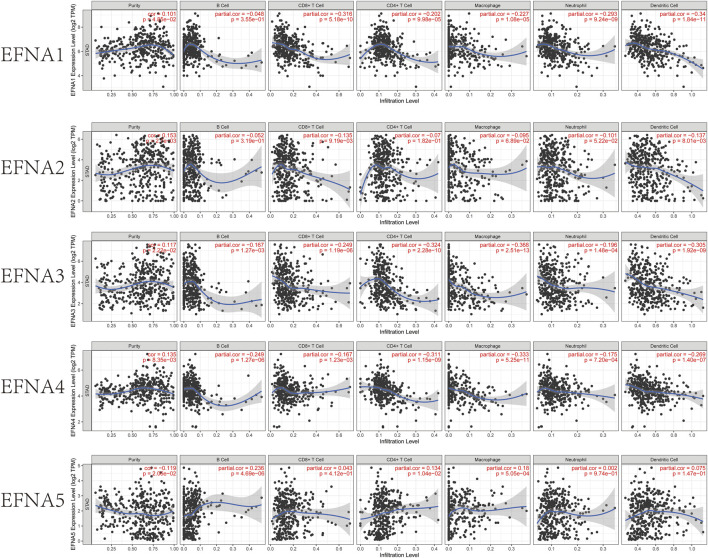
TIMER estimation of the immune infiltration level associated with EFNA1–5 genes. The infiltrating immune cells include B cells, CD8^+^ T cells, CD4^+^ T cells, M2 macrophages, neutrophils, and dendritic cells. Correlation between EFNA1-5 with the abundance of various immune cells in gastric cancer.

In this study, the TIMER database was used to explore the relationship between *EFNA* expression and immune cell infiltration Figure 5. *EFNA1* expression was negatively associated with infiltration of CD8^+^ T cells (Cor = −0.316, *p* = 5.18E-10), CD4^+^ T cells (Cor = −0.202, *p* = 9.98E-05), macrophages (Cor = −0.227, *p* = 1.08E-05), neutrophils (Cor = -0.293, *p* = 9.24E-09) and dendritic cells (Cor = −0.34, *p* = 1.84E-11). The expression of *EFNA2* was negatively correlated with CD8^+^ T cells (Cor = −0.135, *p* = 9.19E-03) and dendritic cell infiltration (Cor = −0.137, *p* = 8.01E-03). The expression of *EFNA3* was significantly negatively correlated with B cells, (Cor = −0.167, *p* = 1.27E-03), CD8^+^T cells (Cor = −0.249, *p* = 1.19E-06), CD4^+^T cells (Cor = −0.324, *p* = 2.28E-10), macrophages (Cor = −0.368, *p* = 2.51E-13), neutrophils (Cor = −0.196, *p* = 1.48E-04), and dendritic cells (Cor = −0.305, *p* = 1.92E-09). Similarly, *EFNA4* expression was negatively associated with B cells (Cor = −0.249, *p* = 1.27E-06), CD8^+^ T cells (Cor = −0.167, *p* = 1.23E-03), CD4^+^ T cells (Cor = −0.311, *p* = 1.15E-09), macrophages (Cor = −0.333, *p* = 5.25E-11), neutrophils (Cor = −0.175, *p* = 7.20E-04) and dendritic cells (Cor = −0.269, *p* = 1.40E-07). Different from the previous four genes, the higher the expression of *EFNA5*, the higher the abundance of B cells (Cor = 0.236, *p* = 4.69E-06), CD4^+^ T cells (Cor = 0.134, *p* = 1.04E-02) and macrophages (Cor = 0.18, *p* = 5.05E-04).

**FIGURE 6:**
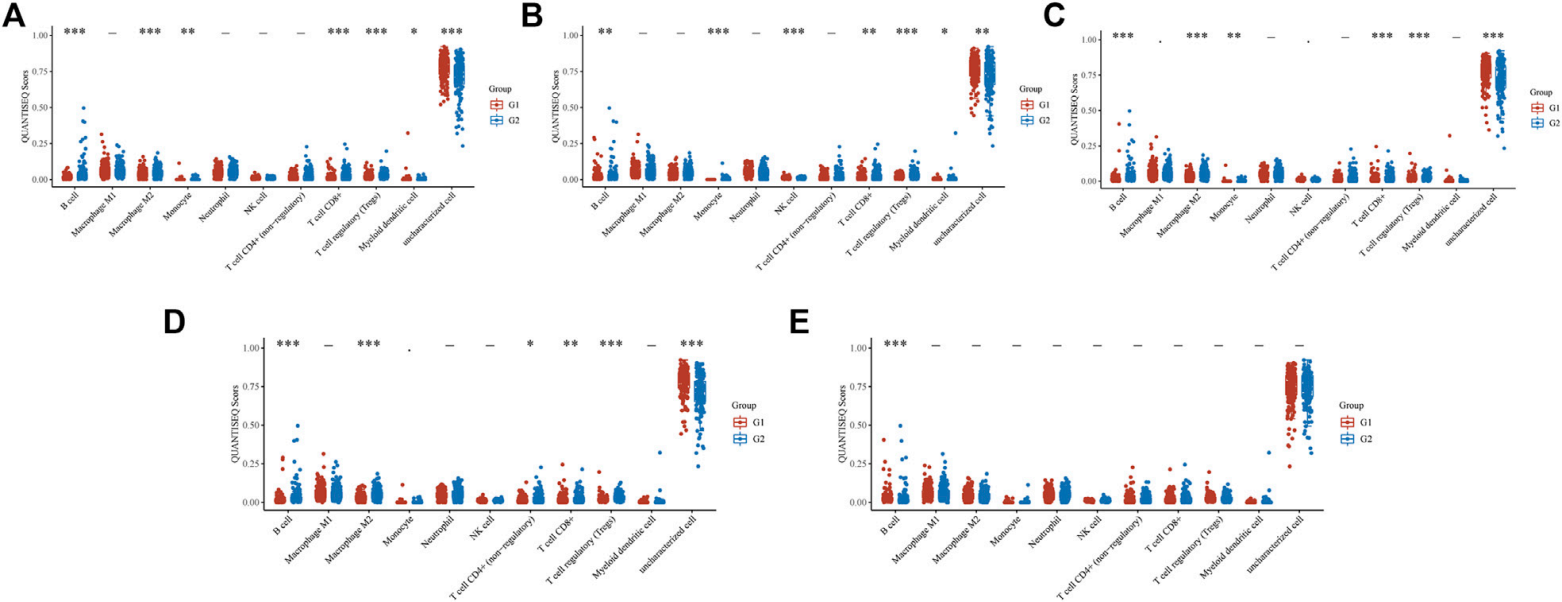
The QUANTISEQ Score distribution of immune cells at different *EFNA* gene expressions. **(A) *EFNA1***, **(B) *EFNA2***, **(C) *EFNA3***, **(D) *EFNA4***, and **(E) *EFNA5***. The horizontal axis represents different immune cells, the vertical axis represents the gene expression distribution, and the different colors represent different groups. Asterisks represent levels of significance (* ***p*** < 0.05, ** ***p*** < 0.01, *** ***p*** < 0.001).

We also used the ‘immunedeconv’ R package to explore the relationship between the *EFNA* family and TIME (Figure 6). The expression of *EFNA1* (Figure 6A) was related to the level of B cells (*p* < 0.001), M2 macrophages (*p* < 0.001), monocytes (*p* < 0.01), CD8^+^ T cells (*p* < 0.001), regulatory T cells (Tregs) (*p* < 0.001), and myeloid dendritic cells (*p* < 0.05). The expression of *EFNA2* (Figure 6B) was related to the level of B cells (*p* < 0.01), monocyte (*p* < 0.001), natural killer (NK) cells (*p* < 0.001), CD8^+^ T cells (*p* < 0.01), Tregs (*p* < 0.001), and myeloid dendritic cells (*p* < 0.05). The expression of *EFNA3* (Figure 6C) was related to the level of B cells (*p* < 0.001), M2 macrophages (*p* < 0.001), monocytes (*p* < 0.01), CD8^+^ T cells (*p* < 0.001), Tregs (*p* < 0.001). The expression of *EFNA4* (Figure 6D) was related to the level of B cells (*p* < 0.001), M2 macrophages (*p* < 0.001), non-regulatory CD4^+^ T cells (*p* < 0.05), CD8^+^ T cells (*p* < 0.01), and Tregs (*p* < 0.001). The expression of *EFNA5* (Figure 6E) was only related to the level of B cells (*p* < 0.001).

### Relationship Between *EFNA* Genes Expression and TME, as Well as the StromalScore in Patients With Pan-Cancer

This study showed that *EFNA* genes expression was significantly positively or negatively correlated with the StromalScore (Supplementary Figure S2A), ImmuneScore (Supplementary Figure S2B) and ESTIMATEScore (Supplementary Figure S2C) of pan-cancer. Similarly, *EFNA* genes expression was also associated with DNA-ss (Supplementary Figure S2D) and RNA-ss (Supplementary Figure S2E) in various cancers.

### Relationship Between *EFNA1-5* Expression and Immune Subtypes, TME and Stem Cell Index in Pan-Cancer and Gastric Cancer

**FIGURE 7:**
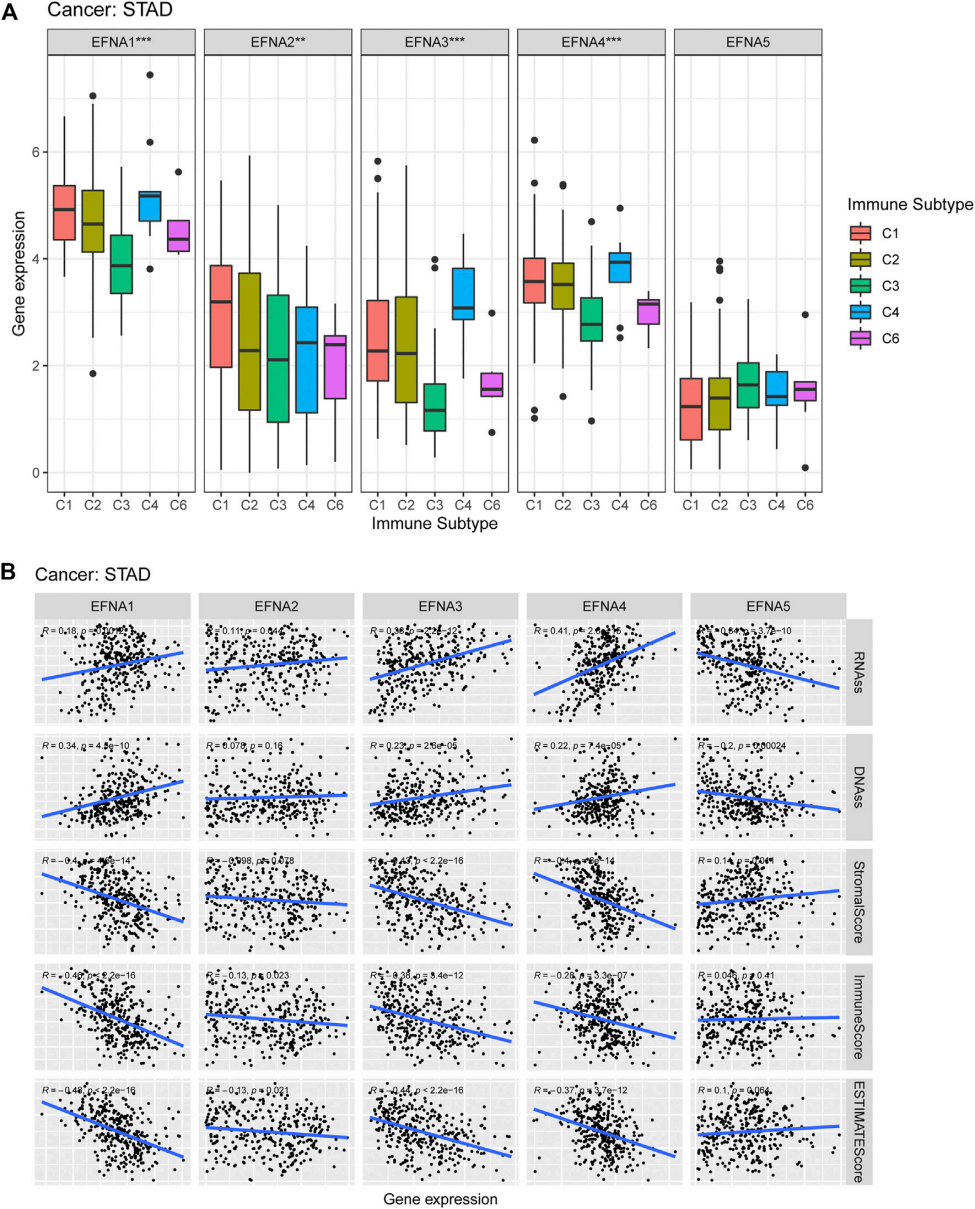
Expression of *EFNA* genes in different immune subtypes, as well as its correlation with the tumor microenvironment and stem cell index. **(A)** Expression levels of *EFNA1-5* in different immune subtypes of gastric cancer. **(B)** Association between *EFNA1-5* expression and RNA-ss, DNA-ss, StromalScore, ImmuneScore and ESTIMATEScore in gastric cancer.

We also investigate the potential correlation between *EFNA* gene expression and immune subtypes in pan-cancer and gastric cancer. *EFNA1-5* showed a significant association with the immune subtype in pan-cancer (*p* < 0.001, Supplementary Figure S3). Figure 7A shows that the expression of *EFNA1-4* in gastric cancer was significantly correlated with immune subtypes (*p* < 0.001, *p* < 0.01, *p* < 0.001, and *p* < 0.001, respectively). *EFNA1-4* was highly expressed in C4. while *EFNA1* was highly expressed in C1–C4, and C6. Elevated *EFNA2* expression was associated with C1 infiltration.

Figure 7B shows that in gastric cancer, *EFNA5* was negatively correlated with RNA-ss (*R* = −0.34, *p* = 3.7E-10) and DNA-ss (*R* = −0.2, *p* = 0.00024), and positively correlated with StromalScore (*R* = 0.14, *p* = 0.011). The expression of *EFNA1-4* was positively correlated with RNA*-*ss (*R* = 0.18, *p* = 0.0012; *R* = 0.11, *p* = 0.044; *R* = 0.38, *p* = 2.2E-12; *R* = 0.41, *p* = 2.8E-15, respectively). Furthermore, the expression of *EFNA1* (R = 0.34, *p* = 4.5E-10), *EFNA3* (R = 0.23, *p* = 2.6E-05), and *EFNA4* (R = 0.22, *p* = 7.4E-05) were positively correlated with DNA-ss. In terms of StromalScore, *EFNA1* (*R* = −0.4, *p =*4.6E-14), *EFNA3* (*R* = −0.43, *p* = 2.2E-16) and *EFNA4* (*R* = −0.4, *P* = 6E-14) showed negative correlation. The expression of *EFNA1-4* was negatively correlated with ImmuneScore (*R* = −0.46, *P* =<2.2E-16; *R* = −0.13, *p* = 0.023; *R* = −0.38, *p* = 3.4E-12; *R* = −0.28, *p* = 3.3E-07, respectively). Similarly, *EFNA1-4* expression was negatively correlated with ESTIMATEScore (*R* = −0.48, *P* = <2.2E-16; *R* = −0.13, *p* = 0.021; *R* = −0.44, *p* = 2.2E-16; *R* = −0.37, *p* = 3.7E-12, respectively).

### Relationship Between *EFNA1-5* and Immune Checkpoints

**FIGURE 8:**
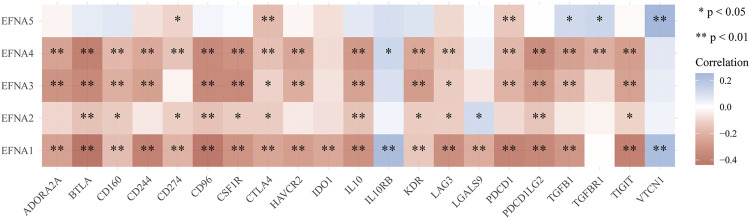
Heat map of correlation analysis between *EFNA* family genes and immune checkpoints. The horizontal and vertical coordinates represent genes, in which different colors represent correlation coefficients (blue represents positive correlation and red represents negative correlation). The darker the color, the stronger the correlation between them; *p < 0.05, **p < 0.01.

The multi-gene correlation hotspot map showed that *EFNA* family genes were significantly associated with multiple immune checkpoints (Figure 8). *PDCD1* was significantly correlated with *EFNA1* (*p* < 0.001), *EFNA3* (*p* < 0.001), *EFNA4* (*p* < 0.001), and *EFNA5* (*p* < 0.001). The higher the expression of *EFNA1* (*p* < 0.001), *EFNA2* (*p* < 0.001), *EFNA3* (*p* < 0.001), and *EFNA4* (*p* < 0.001), the higher the expression of *PDCD1LG2*. *CD274* was significantly correlated with *EFNA1* (*p* < 0.001), *EFNA2* (*p* < 0.05), *EFNA4* (*p* < 0.001), and *EFNA5* (*p* < 0.05). *CTLA4* was positively correlated with *EFNA1* (*p* < 0.001), *EFNA2* (*p* < 0.05), *EFNA3* (*p* < 0.05), *EFNA4* (*p* < 0.001), and *EFNA5*(*p* < 0.001).

### Correlation Between *EFNA* Genes With MSI and TMB

**FIGURE 9:**
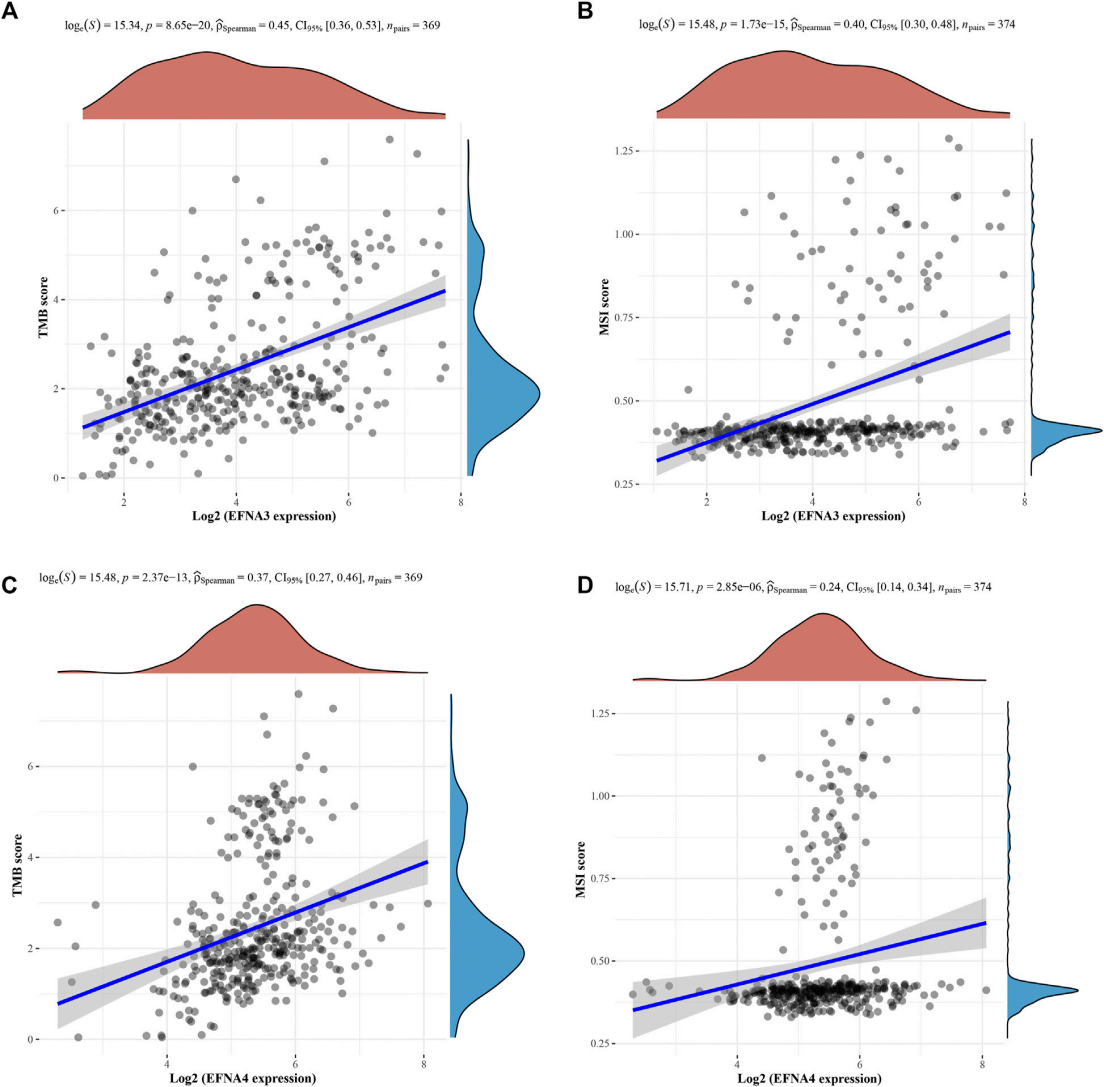
Spearman correlation analysis of TMB/MSI and *EFNA* gene expression. The horizontal axis represents *EFNA* gene expression, and the vertical axis represents TMB/MSI score distribution. On the upper side is the red density curve showing the distribution trend of *EFNA* genes. On the right is a blue density curve showing trends in TMB/MSI fractions. **(A)** *EFNA3* and TMB **(B)** *EFNA3* and MSI **(C)** *EFNA4* and TMB **(D)** *EFNA4* and MSI.

We further explored the association between TMB and MSI and *EFNA* genes expression using Spearman correlation. The analysis results of Figures 9A,B respectively show that the TMB score (*p* = 8.65E-20; 0.45, CI:0.36–0.53) and MSI (*p* = 1.73E-15; 0.40, CI:0.30–0.48) was significantly positively correlated with the expression of *EFNA3*. This correlation was also reflected in *EFNA4*. The higher the expression level of *EFNA4*, the higher the TMB score (Figure 9C, *p* = 2.37E-13; 0.37, CI:0.27–0.46) and MSI (Figure 9D, *p* = 2.85E-06; 0.24, CI:0.14–0.34).

## Discussion

Immune checkpoint inhibitors are promising strategies for cancer treatment, which are aimed at blocking the invasion of tumor cells to the host immune system and stimulating the immune system’s response to tumor antigens, thereby killing cancer cells (Zhang and Chen, 2018; Han et al., 2020; Wei Q. et al., 2021). The mechanism of tumor development is closely related to the immune system, especially within the TME (Oya et al., 2020). The concept of the TME reveals that tumor formation is not simply abnormal cell proliferation but highly organized and complex (Fu et al., 2021). At present, immunotherapy for gastric cancer targets patients with advanced HER-2 -positive status with only a few people benefiting from immunotherapy (Zhang et al., 2021). This prompted our research into more targeted and individualized immunotherapy in the gastric cancer population to maximize the benefits of patients.

A recent study quantified the TME to construct a scoring system for predicting the response of gastric cancer to immune checkpoint inhibitors (Zeng et al., 2021). Li et al. identified six target genes of gastric cancer by bioinformatics and found that they were associated with the TME score (Li Y. et al., 2020). The TME is associated with a key transcription factor that is frequently up-regulated in gastric adenocarcinoma which may beneficial for prognosis (Liu et al., 2020). Liu et al. constructed a gastric cancer prognostic scoring system based on several genes closely related to gastric cancer progression. There were differences in the TME immune score, stromal score and inhibitory immune checkpoint expression between high- and low-risk groups (Liu et al., 2021). In another study on the TME, the prognostic power of tumor-stromal ratio in gastric cancer was no less than that of the TNM stage (Peng Q et al., 2018). Furthermore, Li et al. evaluated the prognosis of major stromal and immune cells in gastric cancer and showed that the abundance of NK cells and stroma plays a role in selecting individuals who would benefit from chemotherapy for gastric cancer (Li B. et al., 2020).

In our study, we explored the association between the *EFNA* genes and the infiltration of immune cells. The expression of *EFNA1* was negatively associated with the infiltration of CD8^+^T cells, CD4^+^T cells, macrophages, neutrophils, and dendritic cells. The expression of *EFNA2* was negatively associated with the infiltration of CD8^+^T cells and dendritic cells. High *EFNA3* expression usually indicated low immune cell infiltration. *EFNA4* expression was statistically correlated with the above immune cells. The higher the expression of *EFNA5*, the higher the abundance of B cells, CD4^+^T and macrophages. We further explored and discussed the TME. *EFNA1*, *EFNA3* and *EFNA4* showed a negative correlation with the stromal score and immune score. High expression of *EFNA2* often suggested a low immune score, but no statistical correlation was found with the stromal score. In contrast, *EFNA5* was positively associated with the stromal score, without showing a positive correlation with the immune score.

MSI is an important concern in gastric cancer. Patients with resectable gastric cancer and microsatellite instability tend to have a better prognosis than patients with microsatellite stability (Puliga et al., 2021). MSI accounts for 8–37% of gastric cancer, which is relatively high (Miceli et al., 2019; Rodriquenz et al., 2020). The results of a meta-analysis involving 21 studies demonstrated a favorable prognosis for patients with gastric cancer and MSI (Polom et al., 2018). Moreover, a bioinformatics study systematically analyzed 271 patients with gastric cancer. In terms of prognosis, the MSI subtype was superior to the microsatellite stable subtype, and this advantage was more significant in the Chinese population (Cai et al., 2020). Ma et al. established a prognostic marker of gastric cancer based on 11 TMB differential genes and found that high TMB may promote immune infiltrate, and patients with high TMB showed a better prognosis (Ma et al., 2021). Baseline tumor burden factors, such as the sum of maximum tumor size and target lesion size, can be used in combination with TMB to evaluate the efficacy of immune checkpoint inhibitors in advanced gastric cancer (Wei X.-L. et al., 2021). In a retrospective analysis of 63 patients with advanced gastric cancer treated with immunotherapy, evidence suggests that PD-L1, CPS, EBV, MSI, and TMB are effective in survival outcomes (Kim et al., 2020). Our study found that TMB score and MSI was positively correlated with the expression of *EFNA3* and *EFNA4* in gastric cancer.

Cyclin-dependent kinase 5 (CDK5) is a member of the protein kinase family that has been shown to play a role in cancer development and the TME (Do and Lee, 2020). Abnormal activation of CDK5 affects the development of triple negative breast cancer. In contrast, inhibition of CDK5 may reduce stem transformation, reverse the immunosuppressive microenvironment, and add a good approach to anti-PD-1 therapy (Bei et al., 2020). In an animal study using the CRISPR-Cas9 genome editing system, PD-L1 was attenuated by specifically knocking out CDK5 to enhance host anti-tumor immunity (Deng et al., 2020). In our study, analyzing the interaction of *EFNA1–5* with the protein network showed that CDK5 was correlated with *EFNA* genes.

The extensive involvement of *EFNA1* in the pathogenesis of tumors has been verified by increasing reports. A microarray analysis combined with basic experiments showed that *EFNA1* and GMAN were associated with the invasion ability of gastric cancer cells (Zhuo et al., 2019). In a study of 222 patients with gastric adenocarcinoma that underwent gastrectomy, immunohistochemical analysis of the samples showed that *EFNA1* expression suggested a poor disease-specific survival benefit (Miyazaki et al., 2013). However, the results of the survival analysis in our study did not show a difference in gastric cancer survival between the high and low *EFNA1* expression groups. This may be due to the differences in our survival assessment indicators and samples. One study, involving 525 gastric cancer samples and 501 controls, found that rs12904 polymorphism in the *EFNA1* gene was strongly associated with gastric cancer risk (Li et al., 2014). In a study using RT-PCR to identify the expressions of *EPHA2* and *EFNA1* in gastric cancer tissues and cell lines compared to normal tissues. *EPHA2* expression was higher in 55% of gastric cancer specimens than in the normal group, and 57% of them were overexpressed—suggesting that the expression of these two genes may be related to the behavior of gastric cancer (Nakamura et al., 2005). Our study also found that *EFNA1* expression was significantly higher in gastric cancer than in normal tissues. Classification and analysis of cancer types showed that *EFNA1* was up-regulated in many tumors, most notably in BLCA. A recent case-control study found that genotype frequency of the *EFNA1* rs4971066 polymorphism was associated with susceptibility to gastric cancer (Pu et al., 2021). Another study also showed that *EFNA1* knockout in gastric cancer cell lines, reduced its invasion and metastasis in mice (Zhuo et al., 2019). The results of immune subtype analysis showed that *EFNA1* was significantly correlated with the immune subtype. Among the queried gastric cancer samples, the samples with changes in *EFNA1* accounted for 8%, and the main gene changes were the enhancement of mRNA expression.

A recent study revealed that *EFNA3* has the potential to become a new target for oral cancer treatment through molecular biology techniques and xenotransplantation models (Wang et al., 2020). Upregulation of *EFNA3* in patients with breast cancer has been associated with shorter metastasis-free survival (Gómez-Maldonado et al., 2015). Bioassay studies demonstrated that *EFNA1*, *EFNA3*, and *EFNA4* expression were higher in breast cancer than in normal tissues, while *EFNA5* showed an opposite trend. High expression of *EFNA4* often reveals poor OS and recurrence-free survival in breast cancer (Liang et al., 2021). Pei et al. created a *SERPINE1*-and *EFNA3*-based hypoxia risk index for gastric cancer (Pei et al., 2021). In our study, the expression of *EFNA3* in gastric cancer was significantly higher than that of the adjacent tissues. The expression of *EFNA3* was elevated in pan-cancer, and the differential expression heat map of different cancers showed that it was elevated in many tumors, but significantly down-regulated in GBM. Drug sensitivity analysis showed that its expression was significantly positively correlated with the sensitivity of SR16157 and fulvestrant.

In recent years, it has been reported that Mir-645 promotes tumor growth, metastasis, invasion and other malignant biological behaviors in colorectal cancer by targeting *EFNA5* (Li S. et al., 2020). *EFNA5* plays a role in the prognostic effects of chemotherapy in patients with advanced gastric cancer (Liu et al., 2019). *EFNA5* is also a possible therapeutic target in ovarian cancer (Yang et al., 2019). From the results of our analysis, *EFNA5* expression was low in most cancers but elevated in CHOL. Survival analysis showed that the *EFNA5* high expression group showed less survival benefit. *EFNA5* was negatively correlated with the sensitivity of many drugs, but its high expression was correlated with a higher sensitivity for irofulven. Furthermore, the high expression of *EFNA3* and *EFNA4* indicates that it is beneficial for OS and DFS of gastric cancer, while the high expression of *EFNA5* indicates a low survival rate. This may be related to the negative correlation between the expression of *EFNA5* and the other four genes of the *EFNA* family.

There are some limitations in this study. The samples in this study were all from online databases, some of which lacked detailed patient information, such as specific treatment regiments. Second, as a retrospective study, the reliability of the results should be confirmed by a large prospective experimental study.

## Conclusion

This study comprehensively analyzed the expression of *EFNA* genes in gastric cancer as well as its correlation with survival prognosis, immunity, the TME, MSI/TMB, IC_50_ of common chemotherapeutic drugs for gastric cancer and drug sensitivity. Our research is expected to provide a new direction for targeted and immunotherapy of gastric cancer.

## Data Availability

Publicly available datasets were analyzed in this study. This data can be found here: Publicly available datasets were analyzed in this study. The datasets analyzed for this study can be found in the following databases: TCGA (https://cancergenome.nih.gov/), UCSC Xena (https://xenabrowser.net/datapages/), Oncomine (www.oncomine.org), cBioportal (https://www.cbioportal.org/), UALCAN (http://UALCAN.path.uab.edu/), and GeneMANIA (http://www.genemania.org).

## Glossary

AUC: area under curve
BLCA: bladder urothelial carcinoma
BRCA: breast invasive carcinoma
CDK5: cyclin-dependent kinase 5
CHOL: cholangiocarcinoma
CI: 95% confidence interval
DFS: disease-free survival
DNA-ss: DNA stemness score
EFN: Eph/ephrin
Eph: erythropoietin-producing hepatocyte
GBM: glioblastoma multiforme
GDC: Genomic Data Commons
GDSC: Genomics of Drug Sensitivity in Cancer
IC_50_: half-maximal inhibitory concentration
MSI: microsatellite instability
NK: natural killer
OS: overall survival
PPI: protein-protein interaction
RNA-seq: RNA-sequencing
RNA-ss: RNA stemness score
ROC: receiver operating characteristic
TCGA: The Cancer Genome Atlas
TIME: tumor immune microenvironmen
TMB: tumor mutational burden
TME: tumor microenvironment
Tregs: regulatory T cells
ESCA: Esophageal carcinoma
STAD: Stomach adenocarcinoma
UCEC: Uterine corpus endometrial carcinoma
LIHC: Liver hepatocellular carcinoma
LUAD: Lung adenocarcinoma
LUSC: Lung squamous cell carcinoma
KICH: Kidney cancer
HNSCC: Head and neck squamous cell carcinoma
PRAD: Prostate adenocarcinoma
KIRP: Kidney renal papillary cell carcinoma
THCA: Thyroid carcinoma
KIRC: Kidney renal clear cell carcinoma
TGCT: Testicular germ cell tumor
THYM: Thymoma
COAD: Colon adenocarcinoma
READ: Rectum adenocarcinoma
ACC: Adrenocortical carcinoma
CESC: Cervical cancer, Cervical squamous cell carcinoma and endocervical adenocarcinoma
DLBC: Lymphoid neoplasm diffuse large B-cell lymphoma
HNSC: Head and neck squamous cell carcinoma
LAML: Acute myeloid leukemia
LGG: Brain lower grade glioma
OV: Ovarian cancer
MESO: Mesotheliom
PAAD: Pancreatic cancer endocrine neoplasms
PCPG: Pheochromocytoma and Paraganglioma
SARC: Sarcoma
SKCM: Skin cutaneous melanoma
UCS: Uterine carcinosarcoma
UVM: Uveal melanoma
