# Supplementary figures and images for: The Pan-Cancer Crosstalk Between the EFNA Family and Tumor Microenvironment for Prognosis and Immunotherapy of Gastric Cancer
Source: Front Cell Dev Biol. 2022 Mar 2;10:790947. doi: 10.3389/fcell.2022.790947 (PMC8924469; doi:10.3389/fcell.2022.790947)

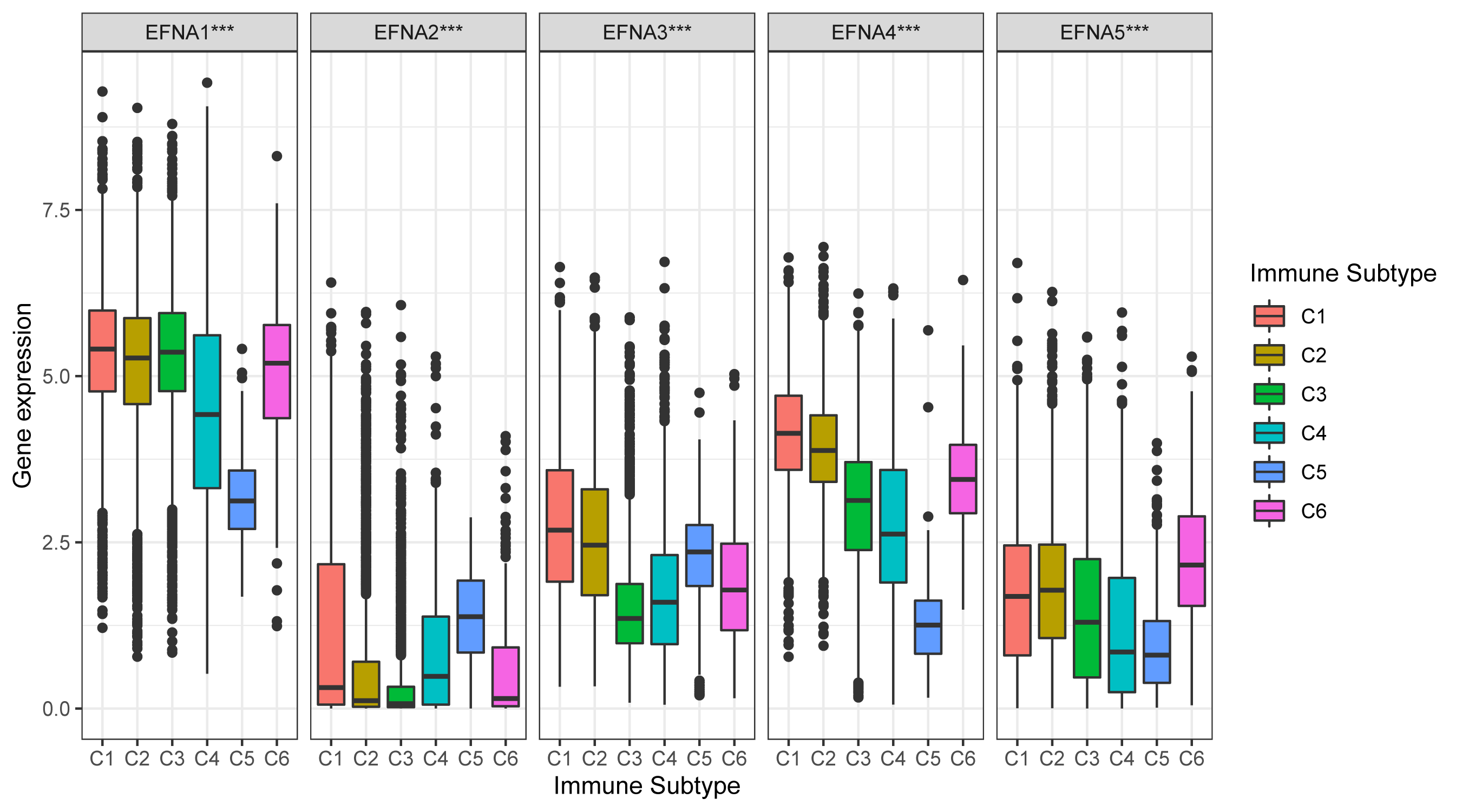

Supplement: Supplementary file 1 [file Image3.TIF]

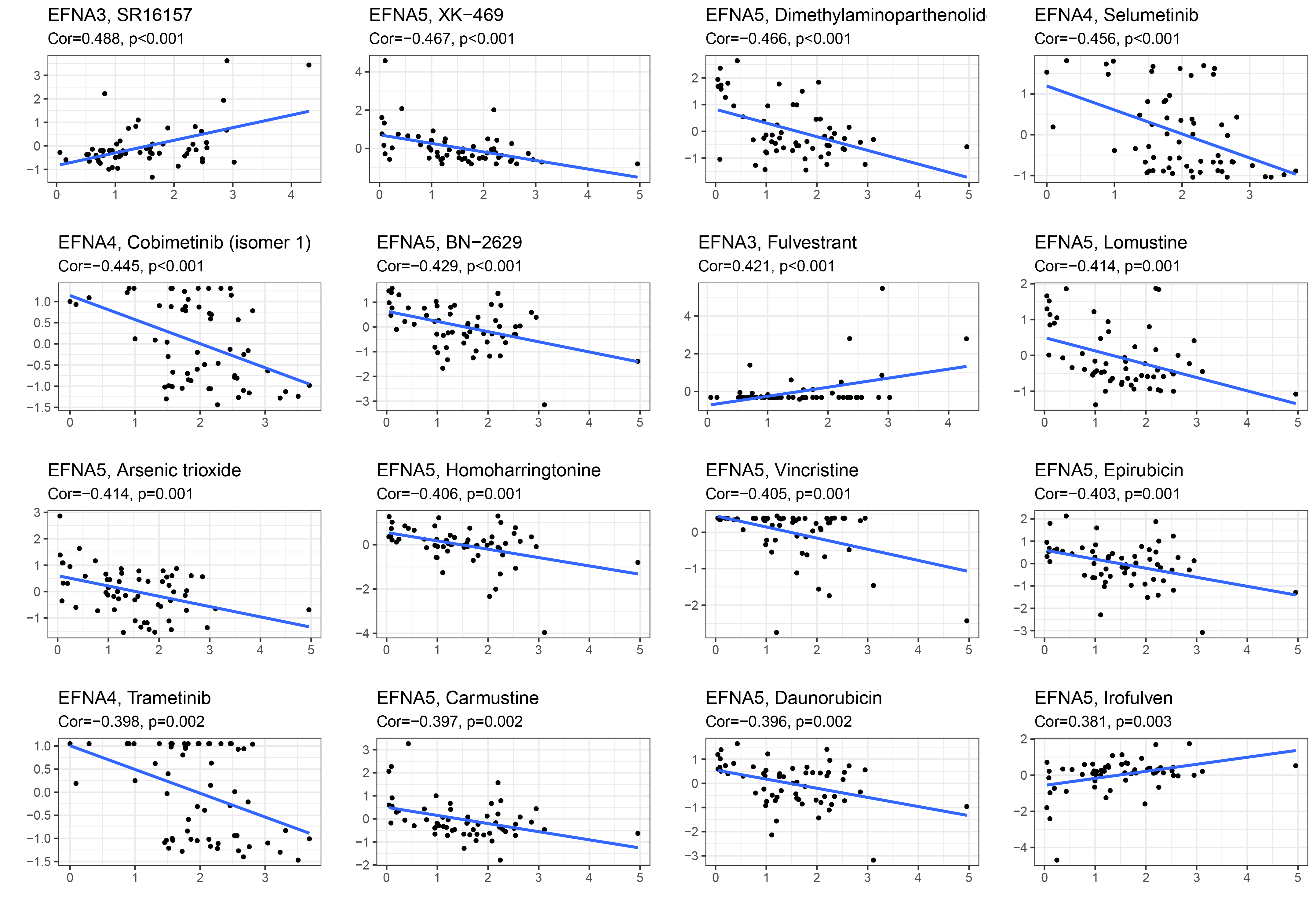

Supplement: Supplementary file 2 [file Image4.TIF]

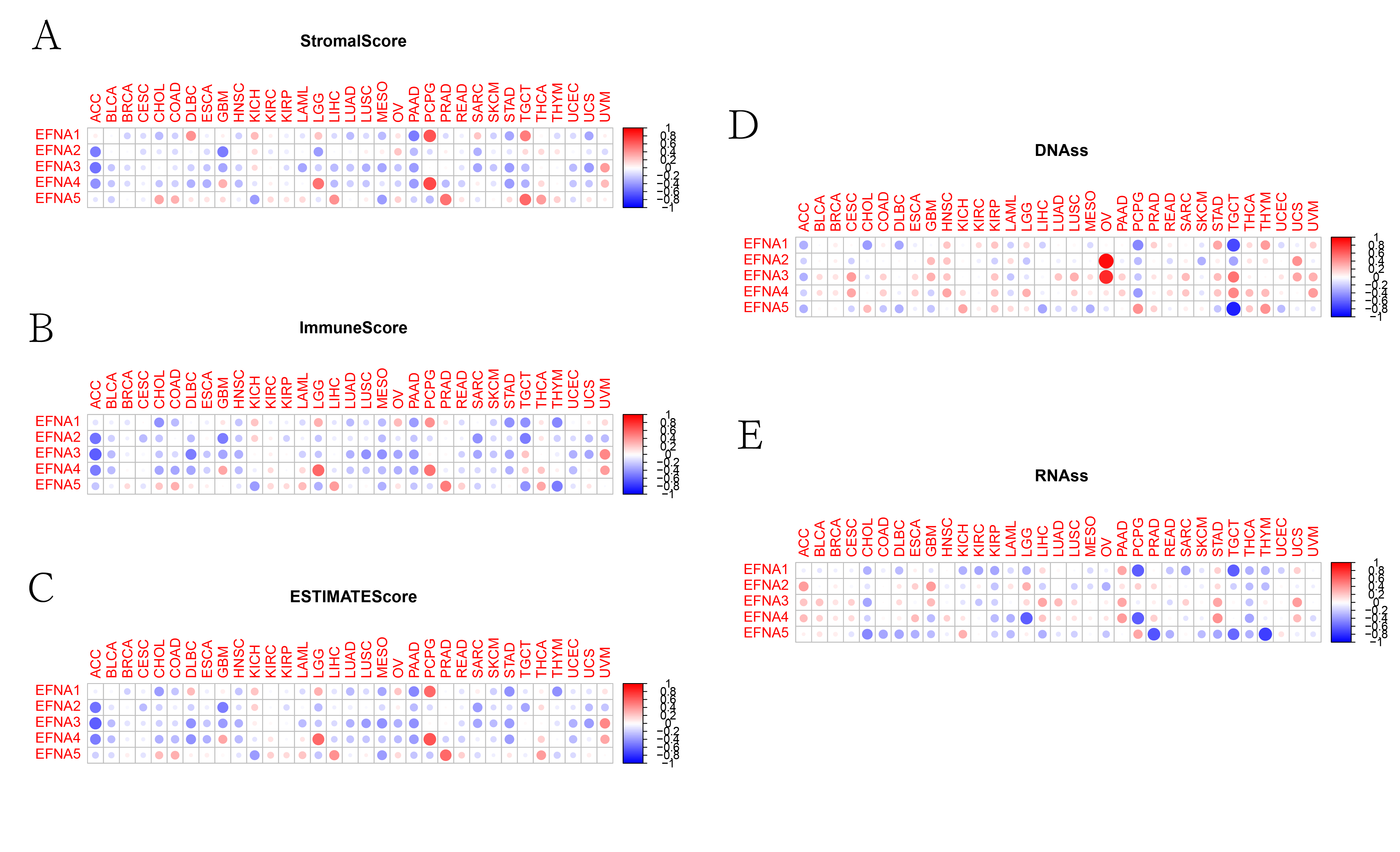

Supplement: Supplementary file 3 [file Image2.TIF]

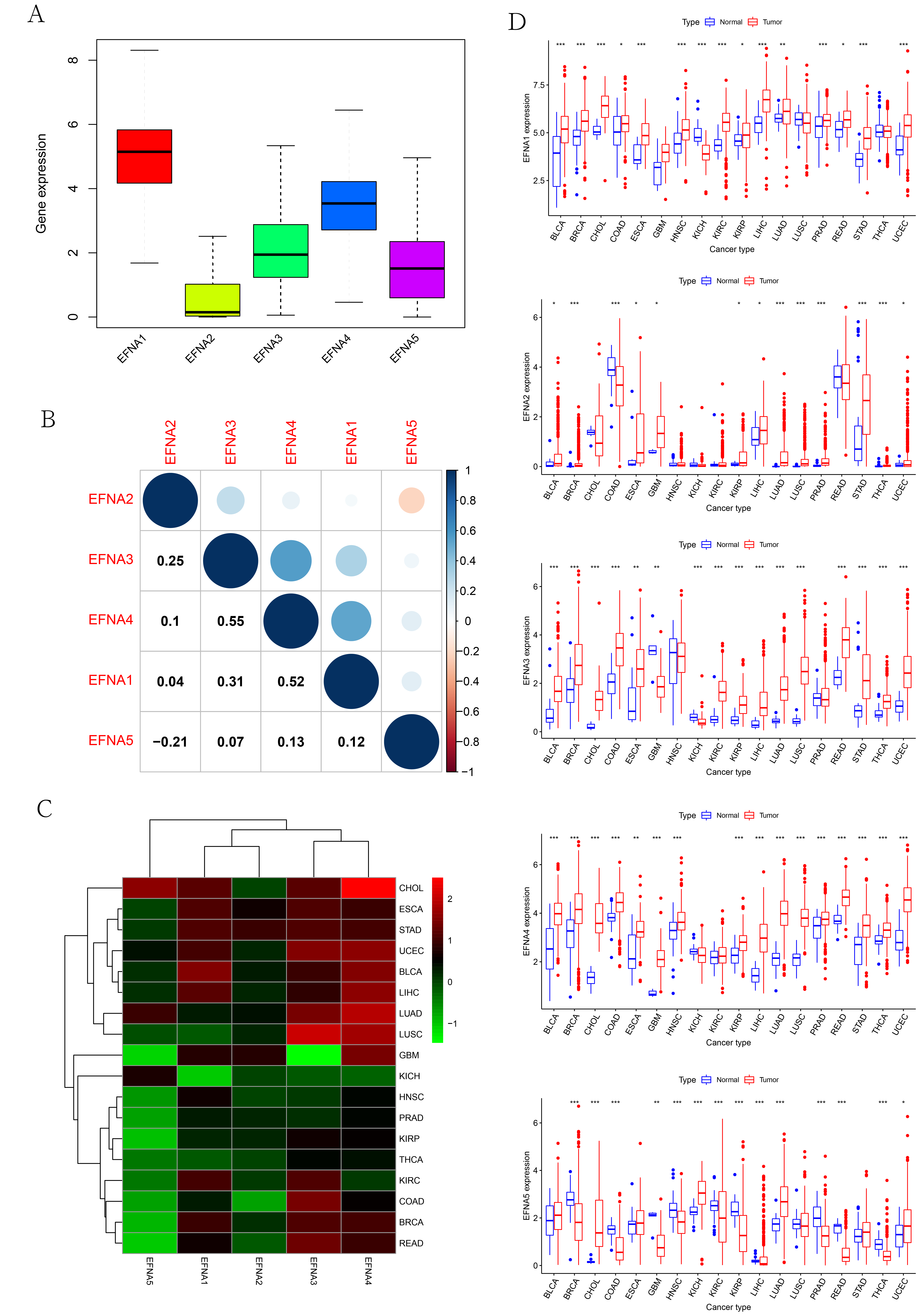

Supplement: Supplementary file 4 [file Image1.TIF]
